# Orodispersible films as a personalized dosage form for nursing home residents, an exploratory study

**DOI:** 10.1007/s11096-020-00990-w

**Published:** 2020-02-13

**Authors:** J. Carolina Visser, Lisa Wibier, Marina Mekhaeil, Herman J. Woerdenbag, Katja Taxis

**Affiliations:** 1 Department of Pharmaceutical Technology and Biopharmacy, University of Groningen, Antonius Deusinglaan 1, 9713 AV Groningen, The Netherlands; 2 Department of PharmacoTherapy, Epidemiology and Economics, University of Groningen, Antonius Deusinglaan 1, 9713 AV Groningen, The Netherlands

**Keywords:** Extemporaneous preparations, Hospital pharmacy, Nursing home residents, Orodispersible films, Personalised medicine

## Abstract

*Background* A frequent problem in ageing patients, and thus in nursing home residents, is dysphagia, affecting the ability to swallow solid dosage forms. A promising and personalized drug delivery system for this patient group is the orodispersible film. Orodispersible films could be prepared extemporaneously in a (hospital) pharmacy setting or in specialty compounding community pharmacies using the solvent casting method. Little has been done to systematically investigate which medications should be chosen for orodispersible film formulation development. *Objective* In this study, the medication use of nursing home residents was examined to identify medications that are suitable for orodispersible film formulation development. *Setting* Nursing homes of three Northern provinces of Netherlands. *Method* Medication intake data from 427 nursing home residents from nine nursing homes from the three northern provinces of the Netherlands were used to identify candidates for orodispersible film formulation development. A stepwise approach, with exclusion steps, was used. Selection criteria included systemic use with a maximum amount of 100 mg per dose unit, no commercially available suitable dosage forms for administration in dysphagia, indication for diseases associated with dysphagia. Furthermore, the characteristics of the active pharmaceutical ingredient needed for the orodispersible film formulation development, such as water solubility and taste, were reviewed. *Main outcome measure* Active pharmaceutical ingredients suitable for orodispersible film formulation development. *Results* The nursing home residents used three hundred forty one different medications. Of those, 34 active pharmaceutical ingredients from six therapeutic groups were considered as candidates for orodispersible film formulation development. Most of these active pharmaceutical ingredients have a bitter taste and poor water solubility, which is a challenge for orodispersible film production. *Conclusions* The most suitable active pharmaceutical ingredient candidates for manufacturing of orodispersible films for the ageing patient population may be the combination of levodopa and carbidopa used to treat the symptoms of Parkinson’s disease, and baclofen used to treat spasticity.

## Impacts on practice

- Pharmacists can compound orodispersible films for patients with special needs for whom commercial available products are unsuitable. Thus, the development of orodispersible films as extemporaneous preparations will contribute to personalized medicine.
- Drug utilization research is an effective tool to explore the most used medications in a patient group. These data can stimulate orodispersible film formulation development.
- The suitability of an active pharmaceutical ingredient for orodispersibe film formulations strongly depends on its characteristics. Not every active pharmaceutical ingredient is suitable to be formulated into an orodispersible film.

## Introduction

Multimorbidity and polypharmacy are very common in older nursing home residents [1, 2]. A frequent problem of ageing is dysphagia, which is associated with a higher risk of mortality. Dysphagia also affects the ability to swallow solid oral dosage forms [3–5]. To overcome this problem, caregivers often manipulate the dosage forms e.g. by dividing or crushing tablets or by opening capsules and mixing the content with food or some liquid [6].

Such adaptations of a dosage form entail a health risk for both patients and caregivers. The functionality of the medication and hence the biopharmaceutical properties can change dramatically, especially in case of modified release products. This may lead to overdosing, efficacy loss, irritation of the stomach and altered absorption in the patient as well as stability problems and bad taste [6]. For caregivers, handling powder when crushing high-risk medications (e.g. lithium) may jeopardize their health. Finally, dosage form adaptations are prone to calculation mistakes. Adequate training in combination with warning symbols [7] reduces erroneous crushing of medications. However, there remains an urgent need for suitable dosage forms in the appropriate dose for special patient groups [6, 8, 9] such as nursing home residents.

A solid dosage form that may facilitate oral drug delivery for these patients is the orodispersible film (ODF). ODFs are placed in the mouth and after disintegration, the medication is swallowed with saliva to enter the gastrointestinal tract. Flexible dosing can easily be accomplished with ODFs, during the manufacturing process or by cutting them into pieces prior to administration [10]. ODFs are already accepted in children [11] to overcome problems associated with swallowing solid oral dosage forms. They have been suggested to be a suitable dosage form for older people [8], especially for patients suffering from dysphagia [12]. For adult use, only a limited number of industrially produced ODFs are on the market, but not available worldwide.

If commercial products are unsuitable or not available or if therapeutic substitution is not feasible, (hospital) pharmacists may compound medications for their own patients. Guidelines are available for the preparation of standardized and non-standardized formulations to ensure reliable products. For example, in The Netherlands a Dutch formulary (Formularium der Nederlandse Apothekers (FNA)) is available with standardized formulations for smaller-scale pharmacy preparations [13]. For the preparation of non-standardized pharmacy formulations, the Royal Dutch Pharmacist Association (KNMP, the professional organization for pharmacists) has developed standardized procedures. These procedures cover the preparation of various dosage forms and describe basic manufacturing processes. They are available on line but no open access. Many of these standardized procedures are incorporated and discussed in the book Practical Pharmaceutics, an international guideline for the preparation, care and use of medicinal products [6, 14, 15]. In view of this, ODFs can be prepared as extemporaneous preparations on a small scale in a (hospital) pharmacy setting or in specialty compounding community pharmacies. Up to now, no standardized formulation is available for ODF preparation. Therefore, the practical applicability and safety aspects (for the patient as well as the compounder) need to be taken into account [6].

Active pharmaceutical ingredients (APIs) should not be hazardous substances and the extemporaneous manufacturing process should be safe and feasible. Different types of hazards are distinguished, from acute hazards (e.g. spilling of strong acids on the skin) to health risk caused by longer-term exposure of APIs (e.g. cancer) [15]. Worldwide several guidelines are available, e.g. from the National Institute for Occupational Safety and Health [16]. In The Netherlands, medications and APIs are classified according to the RiFaS guidelines (Risk assessment for Pharmaceutical substances, Risicoinstrument Farmaceutische Stoffen) [17]. APIs are classified from class 1 to class 5. APIs from class 1 are not harmful (keeping in mind that risk = hazard x exposure) [15] whereas for class 5 APIs special safety measures need to be taken into account. An example in class 5 is any cytostatic drug.

Some characteristics of the APIs may have influence on patient acceptance, such as taste and irritation of the mucosa. Appropriate taste masking is necessary if APIs have a bitter taste [18]. Although the residence time in the mouth is short, an ODF may irritate the tongue and the mucosa, especially if administered repeatedly.

ODFs can be prepared applying a relatively simple preparation method, the solvent casting method. This entails that all excipients are mixed with an aqueous solvent and stirred until a clear or homogeneous solution is obtained. The solution is subsequently cast onto a release liner and dried. The obtained film is cut into the desired size thereby enhancing dose flexibility [10]. In literature, the development of various ODF formulations is described. An example is the development of a low dose enalapril maleate ODF. The formulation contains next to the API, the polymers hypromellose and carbomer 974P and the plasticizer glycerol. Trometamol and disodium EDTA are added to buffer and stabilize the solution in order to make it viscous. After casting, drying and cutting into a size of 1.8 × 1.8 cm the ODF contains 1 mg of enalapril maleate [10]. The amount of excipients per ODF that can be used is limited and usually high potent APIs are incorporated into ODF. However, drug loads up to 50 mg are described in literature [19, 20].

Although, medication use of nursing home residents has been studied widely [21, 22], there has been little attention to use such data as a basis to select candidates for age-appropriate and personalized geriatric medicinal products. In this study, the medication use of nursing home residents was examined to identify medications that are suitable for ODF formulation development, taking into account information on drug utilization and manufacturing-related characteristics of the drugs.

## Ethics approval

The study was approved by the Medical Ethical Committee of the University Medical Center Groningen (Protocol Number NL48091.042.14). Written informed consent was requested from residents or a legal presentative in case of incapability (e.g. dementia).

## Method

Medication use on baseline from a sample of 427 residents (mean age 83.5 years (SD 9.27), 32% male and 68% female), mean number of 7.99 (SD 3.69) medications) from nine nursing homes from the three northern provinces of The Netherlands (Drenthe, Friesland and Groningen) were used. These were data from the Discontinuing Inappropriate Medication Use in Nursing Home Residents (DIM-NHR study). The data was collected between June 2014 and April 2016 as part of a randomized controlled trial on the effects of medication reviews in nursing home residents [23].

The following stepwise approach was used: Firstly, all medications for systemic use with a maximum amount of 100 mg of the active ingredient per dose unit was included. The cut-off of 100 mg was used because the drug load per ODF is limited (to 50 mg per ODF) [19]. An intake of two ODFs of 50 mg at the same time or administration of 50 mg twice daily should be acceptable for the patient. Secondly, the dosage forms of all medications was reviewed and excluded medications where commercial dosage forms in the required dose were available in The Netherlands, suitable for administration in dysphagia (e.g. oral solutions, oral drops, oral suspensions, oral syrups, nasal spray, single dose powders, sublingual tablets and orodispersible tablets). In addition, medications which were only available as modified release dosage forms and medications which were intended for rapid parenteral administration were excluded. Thirdly, the potential indications of the medications was examined. Medications used for symptom control such as pain and medication to treat diseases common in nursing home residents such as cardiovascular diseases, behavioral problems, sleeping disorders and depression were included. Fourthly, the following patient-related characteristics were reviewed: the frequency of use of the medication in the nursing home population, the potential indications for use, the dosages used and the frequency of dysphagia in the disease. Furthermore, the following manufacturing-related characteristics were retrieved from literature for each API: modifications of commercially available oral dosage forms allowed, the taste, hazard class and the water solubility of the APIs. For ODF formulation development water solubility of the API is preferable as the uniformity of content of the ODF is then more easily reached.

## Results

In total, the nursing home residents received 4263 prescriptions corresponding to 341 different medications. Medications used to treat the gastrointestinal tract and metabolism disorders, the central nervous system and cardiovascular disorders were prescribed most frequently. Anti-infective, immunomodulatory, cytostatic and antiparasitic drugs were hardly or not prescribed. In Table 1 the 40 most prescribed medications are listed. The dominant route of administration was the oral route (33 out of 40) and the most prescribed medication was cholecalciferol.

**Table 1:** The 40 most prescribed medications with Anatomical Therapeutical Chemical (ATC) code, administration route and percentage of the nursing home residents receiving this medication

	Medications	ATC code	Administration route	%
1	Cholecalciferol	A11CC05	Oral	61.8
2	Laxative (macrogol/elektrolytes)	A06AD65	Oral	52.1
3	Acetaminophen	N02BE01	Oral	37.7
4	Acetylsalicylic acid	B01AC06	Oral	35.1
5	Esomeprazole	A02BC05	Oral	29.4
6	Omeprazole	A02BC01	Oral	27.5
7	Furosemide	C03CA01	Oral	18.0
8	Emollients and protectives^a^	D02AX	Dermal	15.4
9	Metformin	A10BA02	Oral	15.2
10	Simvastatin	C10AA01	Oral	13.0
11	Calcium/Vitamin D	A12AX	Oral	11.8
12	Cranberry	–	Oral	11.4
13	Metoprolol succinate	C07AB02	Oral	11.1
14	Levothyroxine	H03AA01	Oral	10.6
15	Hypromellose	S01XA20	Oculair	10.0
16	Hydrochlorothiazide	C03AA03	Oral	9.7
17	Calcium carbonate	A12AA04	Oral	9.7
18	Melatonin	N05CH01	Oral	9.5
19	Oxazepam	N05BA04	Oral	9.2
20	Enalapril	C09AA02	Oral	8.3
21	Lactulose	A06AD11	Oral	7.6
22	Fentanyl	N02AB03	Transdermal	7.6
23	Oxycodone	N92AA05	Oral	7.6
24	Dipyridamole	B01AC07	Oral	7.3
25	Temazepam	N05CD07	Oral	7.1
26	Citalopram	N06AB04	Oral	6.9
27	Ferrofumaraat	B03AA02	Oral	6.9
28	Amlodipine	C08CA01	Oral	6.6
29	Prednisolone	H02AB06	Oral	6.4
30	Insulin Glargine	A10AE04	Subcutane	6.2
31	Dermatologicals	^b^	Dermal	6.2
32	Zinc product for dermal use	D02AB	Dermal	5.9
33	Vitamin B12	B03BA03	Intramuscular	5.7
34	Metoprolole tartrate	C07AB02	Oral	5.5
35	Haloperidol	N05AD01	Oral	5.5
36	Digoxin	C01AA05	Oral	5.5
37	Tamsulosin	G04CA02	Oral	5.2
38	Alendronic acid	M05BA04	Oral	5.0
39	Perindopril	C09AA04	Oral	4.7
40	Folic acid	B03BB01	Oral	4.5

^a^Indifferent vehicles, such as hydrophilic creams (cetomacrogol cream and lanette cream, with or without extra petrolatum)

^b^Sudocrem; panthenol ointment; indifferent vehicles and ointments such as petrolatum

**Fig. 1:**
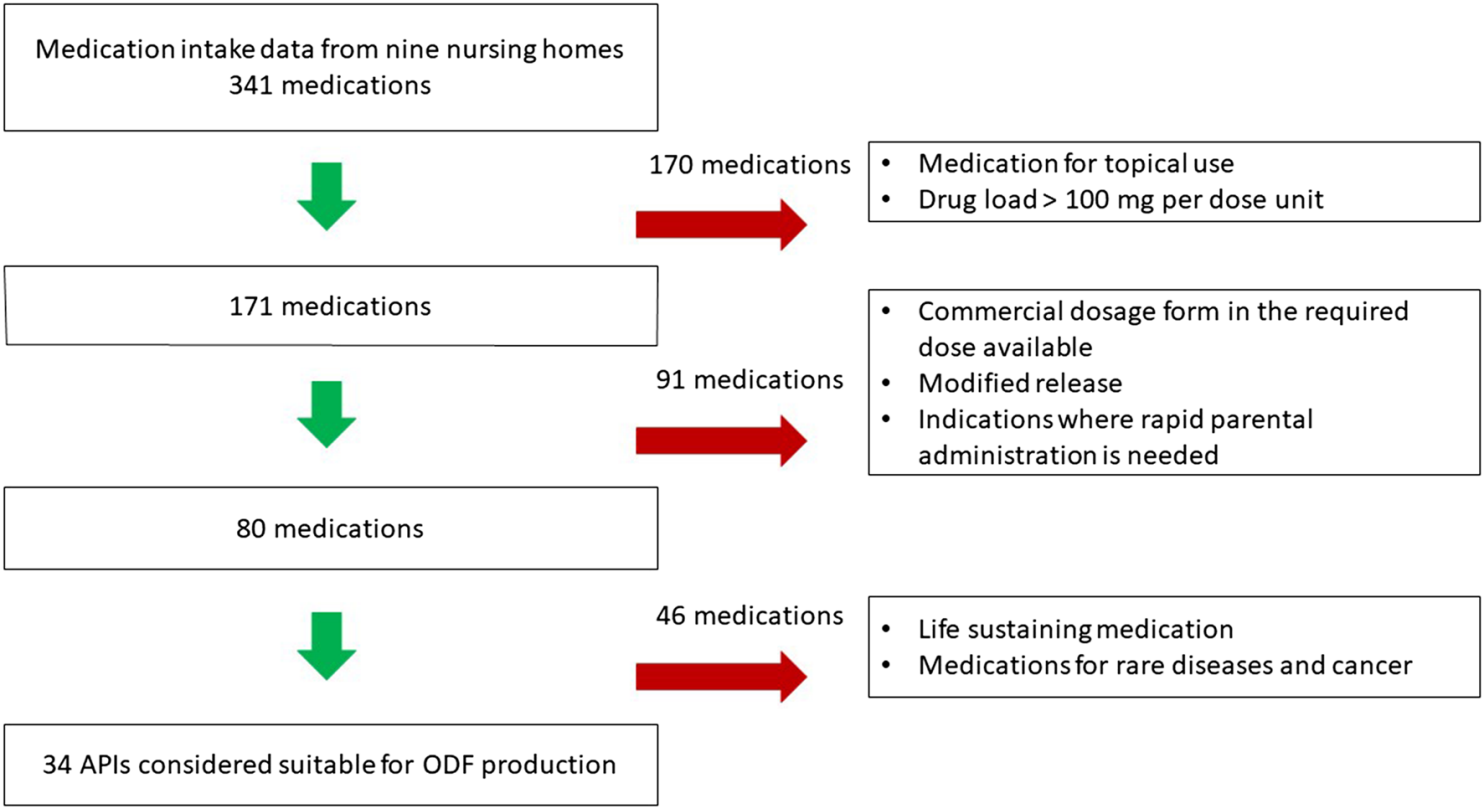
Flowchart for the selection of Active Pharmaceutical Ingredients (APIs) that are suitable for ODF formulation development

In the first step 341 different medications were explored. Hundred seventy medications were excluded in step 1; these medications were for topical use or had a drug load of more than 100 mg per dose unit (see Fig. 1). In step 2, 91 medications were excluded. Of those, there was a commercial and suitable alternative in the required dose available for 52 medications, 14 medications were modified release formulations and 25 medications were intended for rapid parenteral administration. In the third step, 46 medications were excluded as indications were not suitable for ODF administration. Finally, 34 APIs were selected as candidates for ODF formulation development (Table 2). The six ATC main groups to which the selected medications belonged were the gastrointestinal tract and metabolism (ATC A, n = 1); cardiovascular system (ATC C, n = 12); genitourinary system and sex hormones (ATC G, n = 2); anti-infective for systemic use (ATC J, n = 1); musculoskeletal system (ATC M, n = 4); nervous system (ATC N, n = 17). The majority of 34 candidates may be crushed or capsules may be opened, have a bitter taste, are classified in hazard class 1-3 and are poorly water-soluble.

**Table 2:** Characteristics of the 34 selected Active Pharmaceutical Ingredients (APIs) suitable for ODF formulation development

Medications	Patient related characteristics			Manufacturing related characteristics		
ATC-code and name	n*	Indication and uses**^, a^	Modification of the solid dosage form allowed^a^	Hazard class^b^	Taste of the API^c, d^	Water solubility^b^
C01BC04 Flecainide (acetaat)	2	Irregular heartbeat, 50 mg twice a day	Yes May cause irritation of the mucosa	3	–	48.4 mg/mL at 37 °C
C01CA17 Midodrine (HCl)	1	Orthostatic hypotension, 2.5–10 mg three times daily	Yes	–	–	7030 mg/L at 25 °C
C03CA02 Bumetanide	10	Heart failure, 0.5–4 mg once a day	Yes	1	Slightly bitter	> 20 mg/mL (in base)
C03DB02 Triamterene	2	Hypertension, 25–100 mg daily	Yes	1	Slightly bitter	–
C03EA01 Triamterene/hydrochlorothiazide	5	Hypertension, 50/25 mg per day, max 200/100 mg daily	Yes	3	Slightly bitter	–
C07AB03 Atenolol	9	Angina pectoris and hypertension, 50–100 mg daily	Yes, May cause irritation of the mucosa	1	Bitter	13,300 mg/L at 25 °C
C07AB07 Bisoprolol (fumarate)	18	Angina pectoris and hypertension, 5 mg once a day	Yes	1	Bitter	2240 mg/L at 25 °C
C09AA03 Lisinopril (dihydrate)	9	Hart failure (and hypertension), 2.5–35 (80) mg daily	Yes May cause irritation of the mucosa	3	Neutral	97 mg/mL at 25 °C
C09AA04 Perindopril (erbumine)	21	Hart failure (and hypertension), 2–4 (8) mg daily	Yes	2	–	1.22 mg/mL
C09AA05 Ramipril	5	Hypertension and cardiovascular prevention, 2.5–10 daily	Yes	3	Bitter	3.5 mg/L
C09CA06 Candesartan (cilexetil)	4	Hart failure and hypertension, 8–32 mg daily	Yes	3	Neutral	–
G04CA01 Alfuzosin (HCl)	3	Benign prostate hyperplasia, 2.5–5 mg daily	Yes	3	–	92 mg/L at 25 °C
G04CB01 Finasteride	7	Benign prostate hyperplasia, 5 mg daily	No	4	–	11.7 mg/L
J01EA01 Trimethoprim	1	Prevention of bacterial infections (urinary tract), 100 mg daily	Yes	3	Bitter	400 mg/L at 25 °C
M01AH05 Etoricoxib	1	Pain and inflammation (e.g. rheumatoid arthritis), 60 mg daily	Yes	–	Bitter	3.28 mg/L
M03BX01 Baclofen	8	Spasticity, 7.5–20 mg 2–4 times per day	Yes	2	Bitter	2090 mg/L
M03BX02 Tizanidine (HCl)	5	Spasticity, 2–4 mg 3–4 times per day	Yes	1	Slightly bitter	> 20 mg/mL
N04BA02 Levodopa/carbidopa	7	Parkinson’s disease, 100/25 mg 3 times a day	Yes	3	Almost tasteless	5000 mg/L at 20 °C /3.8 mg/mL
N04BB01 Amantadine (HCl)	4	Parkinson’s disease, 100 mg 1 -2 times a day	No	–	Bitter	6290 mg/L
N04BC04 Ropinirole (HCl)	2	Parkinson’s disease, 3–24 mg daily	Yes	2	–	133 mg/mL
N04BC05 Pramipexole (dihydrochloride)	2	Parkinson’s disease, 0.088–3.3 daily	Yes	1	–	3900 mg/L at 25 °C
N04BD01 Selegiline (HCl)	1	Parkinson’s disease, 5–10 mg daily	Yes	1	–	18.2 mg/mL
N05AH02 Clozapine	5	Parkinson’s disease or schizophrenia, 25–300 mg daily	Yes	3		11.8 mg/L
N05BA01 Diazepam	2	Anxiety disorder, 4–80 mg daily	Yes	3	First tasteless, bitter aftertaste	50 mg/L at 25 °C
N05CD01 Flurazepam (HCl)	1	Sleeping disorder, 15–60 mg, usually 30 mg daily	Yes	1	Bitter	500 mg/mL
N05CD02 Nitrazepam	1	Sleeping disorder, 5–10 mg daily	Yes	2	Tasteless	> 42.2 mg/mL
N05CD06 Lormetazepam	2	Sleeping disorder, 1–2 mg daily	Yes	2	–	–
N05CD07 Temazepam	10	Sleeping disorder, 10–40 daily	Yes	2	–	164 mg/L
N05CF02 Zolpidem (tartrate)	2	Sleeping disorder, 10 mg daily	Yes	1	–	23 mg/mL
N06AA04 Clomipramine (HCl)	3	Depression, 50–750 daily	Yes	1	Bitter	0.293 mg/L at 25 °C
N06AA09 Amitriptyline (HCl)	15	Depression, 50–150 mg daily	Yes	3	–	9.71 mg/L at 24 °C
N06AB08 Fluvoxamine (maleate)	4	Depression, 50–100 mg daily	Yes	2	–	–
N06AX05 Trazodone (HCl)	2	Depression, 50–75 mg, 2–3 times a day	Yes	–	Bitter	27.6 mg/L at 25 °C
N06AX21 Duloxetine	1	Depression, 60–120 mg daily	Capsules may be opened	3	–	13 mg/L at 25 °C

*Number of users

**Most frequently used for and uses in target group

^a^[24]

^b^[17]

^c^[25]

^d^[26, 27]

## Discussion

Polypharmacy is very common in nursing home residents. The majority of the medications prescribed in this study were orally administered and used to treat diseases of the gastrointestinal tract and metabolism (e.g. cholecalciferol, laxatives). Furthermore, medications to treat cardiovascular diseases (e.g. furosemide), diseases of the nervous system (e.g. citalopram, haloperidol) and pain medication (e.g. acetaminophen, fentanyl, or oxycodone) were often prescribed. This is in line with other studies examining drug utilization in nursing home residents [2, 21, 22]. Of the 341 different medications used, 34 were identified as suitable candidates for ODF formulation development in a (hospital) pharmacy environment.

As mentioned before, the drug load per ODF is limited to approximately 50 mg API per ODF [19, 20] and an intake of two ODFs of 50 mg at the same time or administration of 50 mg twice daily should be acceptable for the patient. The drug load of an ODF can, however, be increased by increasing the size and thickness of the film or even doubled by the use of a bilayer film. This would imply the intake of one ODF per day, keep in mind that a thick film can negatively influence patient acceptance [28]. For that reason, medications with a drug load higher than 100 mg were excluded. In some cases, the maximum daily dose was a reason for exclusion, for instance for diltiazem. If a patient needs the maximum dose (indication angina pectoris: 360 mg per day) [24], more than seven ODFs per day would be required. The same decision was made for hydroquinone, the maximum dose (indication nocturnal leg cramps: 200 mg during the evening meal and 100 mg before bedtime) [24] would imply six ODFs of 50 mg hydroquinone per day. This is feasible, however not favorable in terms of patient acceptability, especially not in longer lasting therapy. Furthermore, medications with commercially available alternatives for solid oral dosage forms were excluded. It is however important to keep in mind that the administration of larger amounts of oral solutions and oral suspensions might be troublesome, so some medications may still be candidates to be formulated into ODFs.

Up to now, ODF formulation development mainly focusses on immediate release formulations. However, in literature a controlled release system for a mucoadhesive buccal film containing enalapril [29] and a prolonged release of diclofenac from ODFs [30] have been described. The latter research showed that drug-loaded matrix particles can be incorporated in ODFs. The production method of these matrix particles requires sophisticated equipment, which is often not suitable for small scale production. For that reason, medications that were only available as modified release dosage forms, for instance gliclazide or galantamine, were excluded. Finally, medication with indications considered unsuitable for ODF formulations such as cardiac arrest, sepsis or lung embolism, was excluded. In such cases, rapid parental drug administration is required. An example of an API that can only be administered parenterally is darbepoetine alfa, an erythropoietic growth factor. The oral bioavailability of proteins is extremely low and therefore hampers ODF production.

The API characteristics were reviewed and the risk class of the 34 candidates was determined via the RiFaS guidelines [17]. The majority of the candidates fell in classes 1–3 and can be prepared without the requirement of very specific facilities. Water solubility of the API is preferable to reach the uniformity of content more easily. If the API is insufficiently water-soluble, it can be suspended or dissolved with a co-solvent. Many of the 34 candidates are known to be insufficiently water-soluble. An example is alfuzosin, indicated for the treatment of symptoms of benign prostatic hyperplasia, a condition that is frequent in older nursing home resident. The API is water-soluble (92 mg/L) [26] meaning that a co-solvent is needed to incorporate the API into an ODF. Most of the APIs listed in Table 2 have bitter taste and taste masking is needed.

The 34 candidates suitable for ODF formulation development were used for indications commonly present in older patients (e.g. cardiovascular diseases, Parkinson’s disease, benign prostate hyperplasia) or to increase the well-being and quality of life. Medications frequently used were painkillers, anti-psychotics and anti-depressants. Two of the medications (temazepam and perindopril) were among the 40 most prescribed medications others were used less frequently. Such information can be used to estimate the extent of ODF formulation developments needed. The use of the 34 candidate drugs (to be formulated in ODFs) in older patients was discussed with various hospital pharmacist. Although most of the listed medicines may be crushed or capsules may be opened, such manipulations are unwanted [6]. In practice for several medications, therapeutic substitution is feasible. For instance, enalapril solution can be prescribed instead of lisinopril, perindopril, and ramipril tablets. Also, furosemide solution could be prescribed instead of bumetanide tablets. The use of antidepressants as well as benzodiazepines in frail old people should be limited, as there may be a negative benefit risk ratio [31]. Therefore, developing ODFs for those APIs is not a first priority.

An important selection criterion is the frequency of dysphagia in particular diseases. An example is medication for patients suffering from late state Parkinson’s disease. In Table 2 several medications used for Parkinson’s disease are listed. The hospital pharmacist confirmed that ODFs could be an attractive dosage form for patients suffering from late state Parkinson’s disease. The combination of levodopa and carbidopa is the most common and frequently used. The maximum needed dose of 100/25 mg levodopa/carbidopa three times per day would mean an intake of several ODFs per day. The amount of APIs needed for the manufacturing of ODFs exceeds the water solubility. This means that the APIs need to be suspended leading to recrystallization which may result in a gritty surface of the ODF [19, 20]. Usually, this will negatively influence patient acceptance. As late state Parkinson’s disease is associated with severe dysphagia, ODFs might however be a convenient alternative compared to injections [32]. Similarly, the indication spasticity may be another good option as these patients may have severe dysphagia. Baclofen is most frequently used and the maximum dose of 20 mg can be incorporated into an ODF.

## Conclusion

Examining medication use data from nursing home residents taking into account drug utilization and manufacturing related characteristics, we identified 34 APIs candidates potentially suitable for formulation into an ODF for patients suffering from dysphagia. All these candidates can be formulated into ODFs. However, regulatory matters need to be taken into account. If commercial products are available and suitable or if therapeutic substitution is feasible, formulation development is not the first choice. Besides, the API characteristics are important for the selection: the bad (usually bitter) taste should be sufficiently masked and safety measures are needed if the API is potentially hazardous to the compounder. Furthermore, the dose needed for the patient and frequency of dysphagia in certain diseases are important selection criteria.

After using the stepwise approach, it can be concluded that the combination of levodopa with carbidopa and the drug baclofen may be the first candidates for ODF formulation development.
